# A new index of semantic short-term memory: Development and validation of the conceptual span task in Spanish

**DOI:** 10.1371/journal.pone.0209368

**Published:** 2018-12-27

**Authors:** Alaitz Aizpurua, Wilma Koutstaal

**Affiliations:** 1 Psychology Faculty, University of the Basque Country, Donostia-San Sebastián, Gipuzkoa, Spain; 2 Department of Psychology, University of Minnesota, Minneapolis, Minnesota, United States of America; Aix-Marseille Université, FRANCE

## Abstract

Mounting evidence from both cognitive and neuropsychological research points to the importance of conceptual and lexical-semantic contributors to short-term memory performance. Nonetheless, a standardized and well-controlled measure to assess semantic short-term memory was only recently developed for English-speakers, and no parallel measure exists for Spanish-speakers. In the conceptual replication and extension reported here, we develop and validate a Spanish adaptation of the Conceptual Span task as a tool to measure the semantic component of short-term memory. Two versions of the task were validated, the Clustered and the Non-Clustered Conceptual Span task, both in separate samples of 64 and 105 Spanish-speaking university students. We found that both versions of the Conceptual Span task correlate well with another widely used standardized measure of working memory capacity, the Reading Span task. The two versions also correlated, as expected, with discrimination of linguistic congruency as assessed by a semantic anomaly judgment task. Clustered Conceptual Span remained a significant predictor of Reading Span when controlling for several additional cognitive variables, including fluid reasoning, text comprehension, verbal fluency, ideational fluency, and speed of processing. Our results present evidence that the Spanish adaptation of both versions of the Conceptual Span task can yield reliable estimates of the active maintenance of semantic representations in verbal working memory–an under-investigated ability that is involved in diverse domains such as episodic memory retrieval, language processing, and comprehension. Thus, the Conceptual Span task validated here can be employed to predict individual variation in semantic short-term memory capacity in a broad range of research domains.

## Introduction

Short-term memory (STM) and working memory (WM) are fundamental contributors to human cognition, playing a key role in countless aspects of cognitive functioning and general intelligence. Although considerable research has focused on the short-term processing of phonological and visual-spatial information [1], [2], less attention has been devoted to how we represent—and maintain—semantic or conceptual information in short-term memory. This comparative lack of attention has persisted despite increasing evidence of the importance of semantic short-term memory in such diverse domains as language processing and comprehension, analogical thinking, immediate recognition, and episodic memory retrieval. Assessment of semantic STM using a standardized measure, the Conceptual Span task, was first introduced, in an English-language version, over a decade ago [3]. Yet, a parallel index of semantic STM remains unavailable worldwide as an assessment tool for Spanish-speaking participants. In the research reported here, in a conceptual replication and extension of previous work, we develop and validate a Spanish adaptation of the Conceptual Span task as a tool to measure the semantic component of STM.

### The nature and importance of short-term semantic memory

We regard STM, in line with growing empirical findings, as the capacity-limited, activated part of long-term memory [4–8]. Whereas STM specifically refers to “the capacity-limited *retention* of information over a brief period of time," WM refers to "the *manipulation and use* of that information to guide behaviour" (p.1) [5]. Both STM and WM extensively interpenetrate and overlap with other cognitive processes, including long-term memory (LTM). However, research on STM typically focuses primarily on how information is maintained over brief durations whereas research on WM, in addition, considers how that information is "worked with" in memory and adaptively updated during a task. While semantic information contributes to our interpretation of stimuli across multiple modalities (e.g., visual-spatial, auditory, emotional), we here explicitly examine the role of short-term maintenance of semantic information in the processing of verbal information.

The mechanism supporting the active maintenance of phonological representations in verbal WM is well known (i.e., the phonological loop, [1]), and many tasks employed to assess this temporary storage of verbal information (such as, for example, the Digit Span task from the Wechsler Memory Scale-III, [9]) are widely used. In contrast, the memory system underlying the access and holding of lexical-semantic representations has received much less attention; and, consequently, researchers have not shown much interest in the development of instruments to measure the conceptual component of STM.

Nevertheless, there are notable empirical findings within the field of neuropsychology and cognitive psychology supporting a multi-component model that distinguishes separate STM systems for processing of phonological and lexical-semantic information [10–13]. An increasing number of cognitive-behavioral studies have underscored the important contribution of semantic STM to different verbal and nonverbal cognitive abilities, such as language processing and comprehension, analogical thinking, immediate recognition, and episodic memory retrieval [3], [14–19]. For example, in immediate serial and free recall tasks, concrete words are easier to recall than are abstract words [20], [21]. This concreteness advantage is also observed under concurrent articulation (articulatory suppression) that substantially interferes with phonologically-based coding and recall [21], [22], and predominantly reflects a greater likelihood of recalling concrete than abstract items, rather than errors in the ordering of items. A concreteness benefit has also been found under other concurrent attention-demanding tasks that place strong demands on executive resources (e.g., random time interval generation; [23]). Taken together, these and related findings using alternative paradigms and measures (e.g., semantic similarity effects in a fast encoding running span procedure; [24]) argue that the contributions of lexical and semantic knowledge to verbal STM do not depend on controlled and effortful mechanisms of strategic encoding and retrieval, but instead may arise from the automatic encoding of semantic information [23].

In addition, neuropsychological evidence is provided by brain damaged or developmental disorder patients with severe STM impairments that can sometimes predominantly impair either phonological STM or lexical-semantic STM [25–28]. For example, Martin, Shelton, and Yaffee [27] found that a patient with phonological STM impairments possessed largely preserved sentence comprehension abilities but showed sentence repetition deficits, whereas in a patient with impaired semantic STM the reverse pattern was observed, thus showing that phonological processing is minimally involved when only semantic-syntactic reactivation is required. Similarly, Hanten and Martin [29] showed a dissociation between semantic STM and phonological STM in two children with closed-head injuries, with one child showing preserved sentence comprehension (few errors in a sentence anomaly detection task) but impaired sentence repetition (requiring verbatim phonological retention), and the other child showing the reverse pattern (many errors in sentence anomaly detection, but few errors in sentence repetition). More recently, Tan and Martin [19] found that, in nine adults with aphasia, a composite measure of semantic STM was strongly and negatively correlated with accuracy on a semantic interference comprehension task, whereas executive function abilities related to their ability to resolve syntactic but not semantic interference. Additionally, the correlation between performance on the semantic STM composite measure (assessed by category probe recognition and synonymy judgment tasks) and a phonological STM composite measure (assessed by forward digit span and digit matching span tasks) was close to zero suggesting, in line with earlier findings [27], [30], that semantic STM and phonological STM capacities are separable.

### The Conceptual Span task

The Conceptual Span task was first developed by Haarmann, Davelaar, and Usher [3] in order to measure individual differences in semantic STM. One version of the task, the clustered format, presented lists of 12 words, semantically clustered by category, with 4 consecutive words for each of 3 different semantic categories (e.g., *cloud*, *snow*, *drought*, *rain*, *kiwi*, *grape*, *lemon*, *cherry*, *bike*, *truck*, *taxi*, *subway*). In contrast, the second version, the Non-Clustered format, presented lists of 9 randomly ordered words pertaining to 3 different semantic categories (e.g., *hockey*, *nickel*, *parrot*, *tennis*, *rugby*, *owl*, *peso*, *quarter*, *crow*). In both versions of the task, immediately after the list was presented, the participant was asked to recall only the words from one cued semantic category (e.g., *money*), providing the words in any order (i.e., regardless of input order).

In contrast to standard STM tasks (e.g., forward or backward digit span) which require the immediate verbatim repetition of unrelated items in a fixed order, and thus maximize the contribution of phonological rehearsal to performance, the Conceptual Span task requires the immediate recall of words pertaining to one cued *semantic* category, without considering the initial order of presentation of the words. In the Conceptual Span task, the words are presented at a relatively fast rate (one item per second) so as to minimize participant's ability to encode the items into a script-type representation in LTM, thereby more extensively engaging STM [4], [10]. Additionally, participants are asked to read, out loud, all of the words twice directly before the first testing, creating proactive interference that—given the semantic nature of the task together with the length of the presented list—may particularly impact retrieval from LTM [31], [32] and so further promote reliance on STM. Thus this procedure maximizes the involvement of semantic processing, that is, the maintenance and manipulation of *meanings*, and reduces reliance on phonological STM, that is, literal and sequential word repetition.

Notably, whereas the contribution of control processes is likely to be minimal in the phonological storage task (e.g., forward digit span task), the semantic storage task (e.g., conceptual span task) might involve some updating of information as in WM, and perhaps especially so for the Non-Clustered version of the task (see also [33]). For example, using a Non-Clustered version of the Conceptual Span task, Haarmann, Ashling, Davelaar, and Usher [15] found that semantic STM predicted successful performance on a widely-used measure of cognitive control: the AX-Continuous Performance Task [34]. In fact, there is increasing evidence that the *active* maintenance of semantic representations in *verbal* working memory involves attentional capacity [18], [35], although maybe not executive functions [36].

The effective role that semantic STM plays in the human cognitive system was further explored in a study [14], using a modified version of the Conceptual Span task developed by Haarmann and his colleagues [3] that more closely controlled the semantic categories and category names on such dimensions as number of syllables, word frequency, and category size. They found a link between performance on the conceptual span task, flexible remembering (the ability to alternate between recollection of gist and details on demand), and fluid intelligence. Specifically, they found that the Conceptual Span task predicted the ability to successfully retrieve verbatim information to reject semantically similar (but non-experienced) events in an episodic memory task. Across age groups, this measure of episodic specificity modulation was strongly correlated with scores on the clustered version of the Conceptual Span task.

Haarmann and his colleagues [3] also found that the Conceptual Span task (in the Non-Clustered version) predicted individual differences not only in language processing but also in fluid intelligence, showing an important contribution of semantic STM to higher-order cognitive processes. However, in another study [33], it was observed that when the to-be-remembered words were presented in a clustered fashion, the Conceptual Span task lost its ability to predict intelligence compared to an unclustered version, suggesting that the predictive capacity of the Conceptual Span task depends on the ability to reorganize the information (clustering ability, including WM). Nevertheless, in a later study [14] it was found that both versions of the Conceptual Span task strongly correlated with fluid intelligence, although the Non-Clustered version showed a somewhat higher correlation than the Clustered one (*r* (72) = .67, and *r* (72) = .52, for the Non-Clustered and for the Clustered version, respectively, combining across older and younger adults; *r* (36) = .55, and *r* (36) = .22 for younger adults alone; *r* (36) = .61, and *r* (36) = .46 for older adults alone.)

In the research presented here we seek to construct a closely controlled Spanish adaptation of this Conceptual Span task in order to develop an instrument to thoroughly measure individual differences in the semantic component of STM. Spanish is spoken as a first language by more than 427 million people, in 31 countries, and is the second most common language in the world [37]. Developing a Spanish version of the Conceptual Span task could thus meet the needs of a diverse set of international researchers seeking a tool to efficiently assess the role of semantic STM in meaning integration, cognitive control, complex reasoning and problem solving. So as to also examine the role of the reorganization ability, we validated both versions included in the original Conceptual Span task, labelled the Clustered and the Non-Clustered Conceptual Span tasks. Partially paralleling the methods adopted by Haarmann and his colleagues [3], in Study 1 we compare performance on these two newly developed measures with performance on a Spanish adaptation of the Reading Span task [38], originally developed by Daneman and Carpenter [39], and with a modified version of a Spanish-language Anomaly Semantic Judgment Task developed by Ibáñez, López, and Cornejo [40].

Nonetheless, since the sample size for Study 1 (64 participants) was moderate, in Study 2 we assess the correlations of the newly developed measures of Conceptual Span with Reading Span in a further sample, and include additional assessments of cognitive functions, such as fluid reasoning, to further demonstrate the comparability and predictive value of the new Conceptual Span task.

## Study 1

### Materials and methods

#### Participants

Sixty-four Spanish-speaking student volunteers (48 women), with an average age of 20.97 (range 18–32, *SD* = 2.81) from the University of the Basque Country took part in the experiment. They had 13.27 years (*SD* = 1.20) of formal education completed, and rated their subjective state of health as close to excellent on a 5-point scale ranging from 1 (*very poor*) to 5 (*excellent)*, *M =* 4.24, *SD* = 0.74; they also indicated on two 9-point scales that they were in a rather positive emotional state (1 *unhappy*, 9 *happy*), *M =* 6.81, *SD* = 1.15, and moderately activated (1 *calm*, 9 *activated*), *M =* 5.03, *SD* = 1.65. They received monetary compensation for their participation.

#### Materials

*Conceptual Span Task*. A Spanish adaptation of the Conceptual Span Task (in the clustered and in the Non-Clustered versions) was created. The semantic category norms utilized in the original task [41], but updated and translated into Spanish by Aizpurua and Lizaso [42], were used to choose the categories and items (with the exception of the category *earth*, selected from [43]). In order to choose the 12 categories needed (see Table 1 and the S1 File), the following criteria were adopted: (1) the category names must have only two or three syllables, with all the categories employed in [14] meeting this criteria (marked with * in Table 1) being included in the Spanish version; (2) the two versions were broadly matched for the types of semantic categories included (e.g., *birds*, *fruits*, and *clothes* were included in the clustered version, whereas *insects*, *vegetables*, and *body*-*parts* were included in the Non-Clustered version); and (3) the category size (i.e., the number of different exemplars produced within the category), mean responses produced in the category, and exemplar frequency were controlled using norms from [42].

**Table 1:** Category size, mean responses produced in the category, and mean frequency, first position, number of syllables, familiarity, concreteness, and imaginability of the items included in each of the categories in the clustered and the non-clustered versions of the conceptual Span task.

**Clustered**	**Size**	**Mean responses**	**Item frequency**	**Position**	**Syllables**	**Familiarity**	**Concreteness**	**Imaginability**
Birds*	95	5.93	54.38	12.38	2.63	15.48	15.62	15.64
Fruits*	62	8.65	152.00	21.40	2.38	15.66	15.57	15.63
**Clothes**	74	9.00	61.56	9.71	2.63	15.63	15.58	15.64
**Relatives**	56	9.41	325.88	24.93	2.25	15.63	15.57	15.50
Weather*	86	6.06	67.13	7.33	2.38	15.53	15.58	15.62
**Metals**	51	5.03	105.88	17.43	2.13	15.37	15.58	15.35
**Mean**	70.67	7.35	127.81	15.53	2.40	15.55	15.58	15.37
**Non-Clustered**	**Size**	**Mean responses**	**Item frequency**	**Position**	**Syllables**	**Familiarity**	**Concreteness**	**Imaginability**
**Insects**	52	5.26	157.52	14.27	2.75	15.58	15.64	15.63
**Vegetables**	63	5.69	90.25	18.06	2.38	15.50	15.67	15.55
**Body parts**	101	10.09	103.81	15.38	2.13	15.69	15.62	15.61
**Time-units**	45	7.25	152.13	22.40	2.25	15.69	15.50	15.13
Earth*	62	4.46	74.13	20.50	2.88	15.56	15.58	15.49
Trees*	78	6.20	69.19	10.25	2.63	15.31	15.48	15.44
**Mean**	66.83	6.49	107.84	16.81	2.50	15.56	15.58	15.49

Note.

* Categories included in the English version in [14].

For familiarity, concreteness and imaginability, we used log^n^ for the values from the LEXESP norms [44], and round the transformed values to two decimal places.

In addition, in order to select items within the categories for the Spanish format the following criteria were adopted: (1) the items within the categories must each consist of only two or three syllables; (2) the most common item in the category provided in the norms from [42] was excluded; (3) number of syllables, frequency, familiarity, concreteness, imaginability, and first position (i.e., number of times the item was produced as the first in its category) of the selected items were controlled using the LEXESP norms [44], so as to control the possible differences between the Clustered and the Non-Clustered versions of the Conceptual Span task in these aspects (see Table 1; note that for ease of comparison, we use log^n^ for the values from the LEXESP norms).

Differences were not statistically significant between the Clustered versus Non-Clustered versions, respectively, in category size (*M* = 70.67, *SD =* 17.41 and *M* = 66.83, *SD =* 20.13), mean number of responses (*M* = 7.34, *SD =* 1.88 and *M* = 6.49, *SD =* 1.99), item frequency (*M* = 127.81, *SD =* 103.69 and *M* = 107.84, *SD =* 34.43), position (*M* = 15.53, *SD =* 16.81 and *M* = 16.81, *SD =* 4.43), number of syllables (*M* = 2.40, *SD =* 0.20 and *M* = 2.50, *SD =* 0.30), familiarity (*M* = 15.55, *SD =* 13.31 and *M* = 15.56, *SD =* 13.54), concreteness (*M* = 15.58, *SD =* 11.73 and *M* = 15.58, *SD =* 13.01) or imaginability (*M* = 15.57, *SD =* 13.38 and *M* = 15.49, *SD =* 13.69). The S1 File provides the categories and items for the clustered and Non-Clustered versions of the Conceptual Span task.

Thus, the Conceptual Span Task included, as in previous research [3], [14], 12 categories with eight items each (96 items in total). Both versions of the task started with two practice trials. The clustered version presents 16 lists of 12 words, semantically clustered by category, with four consecutive words for each of three different semantic categories (e.g., *hail*, *thunder*, *hurricane*, *wind*, *orange*, *banana*, *strawberry*, *pear*, *stork*, *ostrich*, *sparrow*, *falcon*). In contrast, the second version, the Non-Clustered format, presents 16 lists of 9 randomly ordered words pertaining to three different semantic categories (e.g., *sea*, *minute*, *month*, *chard*, *leeks*, *hour*, *gulf*, *valley*, *onion*). In both versions of the task, on each trial, participants silently read a randomly ordered list of words and, immediately after the list is presented, the participant is asked to recall only the words from one cued semantic category, providing the words in any order (i.e., regardless of input order). Specifically, following the presentation of the last word, the name of one of the three categories appears in capital letters and participants attempt to recall aloud all the words in that category (e.g., hail, thunder, hurricane, wind, orange, banana, strawberry, pear, stork, ostrich, sparrow, falcon, BIRDS? Correct answer: stork, ostrich, sparrow, falcon, given in any order). Their score was the average number of words they could recall out of four words (Clustered version) or three words (Non-clustered version) across a series of such trials.

*Semantic Anomaly Judgment Task*. A modified version of the original tasks developed by [40] was used. Some minor changes were made regarding the Spanish words included in the task (in order to adapt it to our population, because some words in Spanish are not the same in Chile, where the original task was developed, and in Spain; see Table 2 for the specific changes). Three sentences from [40] were used as examples of the task (sentences 2, 8 and 18). Each of the other 24 sentences were presented four times, two with the congruous ending, and two with an incongruous ending (96 trials in total); for the two incongruous endings, one was incongruous with either the first or the second part of the sentence, whereas the other incongruous ending was incongruous with both parts of the sentence (see Table 2).

**Table 2:** List of sentences and endings employed in the semantic anomaly judgment task.

Sentences	Congruent	Incongruent		
		1	2	3
**1. Algo redondo y que da la hora es un**	reloj	volante		grifo
**2. Emite luz y está lejos de la tierra**	sol	foco		sopa
**3. Una prenda de vestir que se pone en las manos es un**	guante	chaqueta		cuadro
**4. Algo cilíndrico que se usa para escribir**	lápiz	tubo		luna
**5. Algo de metal que sirve para cortar el papel es**	tijeras	campana		rosal
**6. pone huevos y tiene plumas es**	gallina		plumero	lámpara
**7. Algo de metal y que sirve para clavar es**	martillo	olla		cuerda
**8. Tiene motor y sirve para movilizarse en las calles, es**	coche	buque		pino
**9. Tiene motor y sirve para transportar cargas, es**	camión	taladro		sillón
**10. Algo que tiene cuatro patas y muerde, es**	perro	mesón		llaves
**11. Algo que tiene cuatro patas y se puede montar, es**	caballo	ratón		lechuga
**12. Tiene cuatro patas y da leche, es una**	vaca	iguana		sombra
**13. Tiene orejas grandes y trompa larga, es un**	elefante	conejo		disco
**14. Es de metal y sirve para freír, es**	sartén		aceite	plumón
**15. Un lugar seco y con mucha arena, es**	desierto	incendio		nubarrón
**16. Tiene olas y es salado, es un**	mar	lago		pasillo
**17. Es verde y se encuentra en las canchas de fútbol, es el**	césped		balón	cinturón
**18. Vuela y tiene un motor, es un**	avión		moto	monte
**19. Un mueble de madera y que se usa para comer en él, es**	mesa		cuchara	orca
**20. Algo que tiene sábanas y sirve para dormir, es**	cama		sedante	víbora
**21. Tiene bisagra y está en la entrada de casa, es**	puerta		jardín	canción
**22. Tiene flores y está sobre la mesa, es un**	florero		tenedor	colegio
**23. Un ave y que vive en la costa es**	gaviota		foca	diario
**24. Algo de color blanco y que endulza es**	azúcar		manjar	grito
**25. Tiene teclas y es un instrumento musical, es un**	piano		violín	zumo
**26. Se bebe y se obtiene de la vaca, es**	leche		carne	barca
**27. Es un mueble y sirve para sentarse, es**	silla		piedra	dedo

*Reading Span Task*. The Spanish adaptation of the task [38], originally developed by [39] was employed. On each trial of this task, participants read aloud a set of sentences, presented one sentence at a time on a display monitor. As soon as the participants finished reading the last word in a sentence, the participant pressed a key that led to the display of the next sentence in the set. At the end of each set a question mark appeared and participants attempted to recall aloud all the sentence-final words in the set in their order of presentation. The set size varied from two to six sentences and there were three trials at each set size. A particular sentence occurred only once in the test, always ended in a concrete noun, and could be from 13 to 16 words long. (An example of a trial at set size 2 is *According to all the surveys Robert Redford is the most famous actor of the cinema*. *That summer was so cold that many people had to change their plans*. Correct answer: cinema, plans.) The Reading Span test started with two practice trials at set size 2 and the actual test began at this set size. Each time a participant answered two trials at a particular set size correctly, the set size was increased by one sentence and participants were instructed that such an increase would take place. Testing was discontinued if a participant answered zero trials correctly at a particular set size. A correct trial was one in which all the sentence-final words in a sequence of sentences were recalled in the correct sequential order. Participant’s score for the Reading Span task was defined as the level at which the participant achieved two correct trials (the minimum possible score was 2 and the maximum possible score was 6).

#### Procedure

All procedures were approved by the Ethics Committee Board of the University of the Basque Country (UPV/EHU). The experiment began with the process of obtaining written informed consent, after which participants completed a brief demographic inquiry form. Then they were tested individually and performed three tests, given in the same order: Conceptual Span, semantic anomaly judgment task, and Reading Span. The Conceptual Span Task was administered first in order to prevent performance on this task from being influenced by learning or interference from the other memory tasks. Also, the clustered version of Conceptual Span task was presented first, because it is the purest measure of short-term semantic memory. All words and sentences in the tasks were visually presented using software E-prime 2.0 [45], in order to present stimuli in a controlled manner and visual format. The experimental session lasted approximately 55 minutes.

### Results and discussion

For all statistical analyses, the significance level was set at *p <* .05, unless otherwise noted.

### Performance on the tasks

Table 3 shows mean performance for each of the tasks: clustered conceptual span (and for each of the separate clusters within this task), Non-Clustered conceptual span, Reading Span, and the semantic anomaly judgment task.

**Table 3:** Mean performance on the measures employed in Study 1.

Measures	Mean	*SD*	Min	Max	Maximum possible
**Clustered Conceptual Span**	40.98	7.83	21	57	64
**Cluster 1**	13	4	5	21	24
**Cluster 2**	15	4	5	22	24
**Cluster 3**	13	2	7	16	16
**Non-Clustered Conceptual Span**	28.80	5.53	16	39	48
**Reading Span**	2.97	0.82	2	5	6
**Semantic anomaly judgment task**	0.98	0.02	0.89	1	1

On the key clustered version of the Conceptual Span Task participants recalled, on average, a proportion of .64 (*M* = 40.98 words out of 64 words maximum; range 21–57, *SD* = 7.83), whereas in the more difficult version they recalled .60 (*M* = 28.80 words out of 48 words maximum; range 16–39, *SD* = 5.53). Thus, participants had a higher proportion of words recalled in the Clustered than in the Non-Clustered version of the Conceptual Span task, *t*(63) = 3.65, *p* = .001. Note that although in [3], for the Clustered version in Study 3 the mean proportion was .73 (*M* = 47, *SD* = 7.93, range 27–60), that is, .11 (7 words) more than in our study, mean proportion in the Non-Clustered version was .60 (*M* = 28.92, *SD* = 5.77, range 13–40) in Study 1 with 66 young adults, and .63 (*M* = 30, *SD* = 5.31, range 19–40), in Study 3 with 64 young adults, very similar to the performance obtained here. (In Aizpurua and Koutstaal [14] with 36 younger adults, mean proportion in the Clustered version was .77 (*M* = 49.08, *SD =* 5.40, range 37–60), and in the Non-Clustered version was .69 (*M* = 33.22, *SD* = 3.86, range 23–39.)

To facilitate across-study comparisons and given that the Conceptual Span task is analogous to the partial report task [46], we also used the same method as earlier adopted by Haarmann and his colleagues [3] to obtain an estimated assessment of semantic STM capacity. Specifically, for each version of the task we found the average number of items recalled per trial × the number of categories that could be probed in a memory list × a conservative guessing correction. Given that 4 out of 8 (Clustered version), and 3 out of 8 (Non-Clustered version) items in a category were probed per trial, and that subjects knew the pool from which words were sampled, for the Clustered version, the guessing correction was 1 minus 4/8, and for the Non-Clustered version the correction was 1 minus 3/8. This yielded an estimated capacity of 3.8 for the Clustered Conceptual Span Task and 3.4 for the Non-Clustered Conceptual Span Task. The latter value is identical to that reported in Experiment 1 of the original study [3] for the Non-Clustered Conceptual Span. Both values are comparable to the estimate of 3 to 5 chunks argued by Cowan [4] as a typical mean STM memory capacity for adult humans.

We used the Spearman-Brown coefficient to calculate the split-half reliability for odd-numbered versus even-numbered trials of the Clustered and Non-Clustered Conceptual Tasks. This indicated satisfactory reliability for both measures: .79 for Clustered and .78 for Non-Clustered.

On the Semantic Anomaly Judgment Task, the proportion of correct responses was on average .98 (*SD* = .02), with a reaction time of 8932.33 ms, *SD* (64) = 18181.32 for correct responses, and a reaction time of 4886.32 ms, *SD* (42) = 6257.37 for incorrect responses. Thus, participants were considerably slower when making correct judgments than when making incorrect judgments in this task. In addition, looking in more detail at the type of ending (i.e., incongruous 1 or 2 vs. incongruous 3 trials), participants had significantly more correct judgments for incongruous 3 (*M* = .99) than for incongruous 1 or 2 (*M* = .97), *t*(63) = 3.18, *p* = .002, and they needed less time to make these correct judgments for incongruous 3 (*M* = 6674.85) than for incongruous 1 or 2 (*M* = 8657.60), *t*(63) = 2.18, *p* = .033. These results are possibly due to the fact that, in comparison with incongruous 1 or 2 endings (i.e., statements for which the first *or* last part of the sentence was semantically incongruous), sentences with incongruous 3 endings were more obviously incongruous and were more easily (and more rapidly) identified as incongruent given that both the first *and* the last part of the sentence were amiss.

Finally, on the Reading Span Task, participants' mean score was 3.02 (maximum score was 6; range 2–5, *SD* = 0.86). This score is slightly lower but highly comparable to the score reported by [3], with 60 young adults (*M* = 3.93, *SD* = .84).

### Correlations between measures

Table 4 shows the product moment correlations between the measures. The Clustered and Non-Clustered versions of the Conceptual Span Task positively correlated with each other, *r*(64) = .73, *p* < .001, with this value being highly comparable to the correlation *r*(64) = .67 observed in [3]. The correlations in [14] were *r*(72) = .77 in older and younger adults, and *r*(36) = .46, *p* < .01 in only young adults; however, note that in [14] the two versions were administered in different testing sessions. Pearson correlation between cluster 1 and 2 was *r*(64) = 0.57, *p* < 0.01, between cluster 1 and 3 was *r*(64) = 0.35, *p* < 0.01; and between cluster 2 and 3 was *r*(64) = .19.

**Table 4:** Pearson correlations between measures employed in Study 1.

Measure	Clustered Conceptual Span	Non-Clustered Conceptual Span	Reading Span	Semantic Anomaly Judgment
**Clustered Conceptual Span**		0.73**	0.55**	0.27*
**Cluster 1 (C1)**	0.88**	0.67**	0.50**	0.29*
**Cluster 2 (C2)**	0.83**	0.54**	0.44**	0.17
**Cluster 3 (C3)**	0.54**	0.44**	0.29*	0.16
**Non-Clustered Conceptual Span**			0.28*	0.24
**Reading Span**				0.21

*p < 0.05.

** *p* < 0.01

As predicted, there was a significant positive correlation between performance on the Reading Span Task and the average score on the Clustered Conceptual Span Task, *r*(64) = .55, *p* < .001, and with the scores on the Non-Clustered Conceptual Span Task, *r*(64) = .28, *p* = .025. Haarmann and his colleagues [3] did not examine the former, but regarding the later they found numerically stronger correlations between the Non-Clustered version and the Reading Span, *r*(64) = .37, *p* < .01 in Study 1, and *r*(60) = .47, *p* < .01 in Study 2.

The correlations between the scores in the Semantic Anomaly Judgment Task and the scores in the Conceptual Span Task were *r*(64) = .27, *p* = .03 for the clustered version and *r*(64) = .24, *p* = .059 for the Non-Clustered version. In this case, Haarmann and his colleagues [3] found in their Study 2 a stronger correlation of *r*(60) = .42, *p* < .001 with the Non-Clustered version.

To more closely examine the relation between performance on the clustered version of the Conceptual Span Task and performance on the semantic anomaly task, we first grouped participants based on their Clustered Conceptual Span performance into three groups––a high conceptual span group (scoring 1 or more SD above the mean, average conceptual span performance of .81, *n* = 13), a low conceptual span group (scoring 1 or more SD below the mean, average of .43, *n* = 8) and a middle conceptual span group (within one SD of the mean, average of .63, *n* = 43). We then ran mixed-factor ANOVAs to compare the mean performance of the three groups on the semantic anomaly judgment task for the different types of items (that is, incongruous 1 vs. incongruous 2 items, and incongruous 1 and 2 vs. incongruous 3).

Although overall accuracy for the incongruous 1 and incongruous 2 items was very high, performance in all three groups showed an increase in errors for the more difficult incongruous 2 items (for which the anomaly occurred toward the end of the sentence) than for the incongruous 1 items (for which the anomaly occurred near the beginning of the sentence): high conceptual span group, means of 1.00 vs. .975; middle, means of 1.00 vs. .95; low, means of .969 vs. .949, leading to a main effect of item type, *F*(1, 61) = 7.20, *p* = .009, no effect of group, *F* < 1.6, and no interaction, *F* < 1. Performing the same analysis on response times showed that response times were significantly slower for the more difficult incongruous 2 items (mean = 6182.53) than for the incongruous 1 items (mean = 5240.38), leading to a main effect of item type, *F*(1, 61) = 5.58, *p* = .021. The increase in response time for the incongruous 2 items was present for each of the conceptual span groups (*increases* of 643ms, 1,333ms, and 851ms for high, middle, and low groups, respectively), with no main effect of group, and no interaction, *Fs* < 1.

Combining incongruous 1 and 2 items, and contrasting performance for these two types of items with the easiest incongruous 3 items (for which an anomaly occurred in both the beginning and the end of the sentence), similarly revealed a main effect of item type, *F*(1, 61) = 5.51, *p* = .022, means of .974 vs. .989 respectively. There was no group × item type interaction, *F* < 1, but a trend toward a main effect of conceptual span group (mean high = .991, mean middle = .984, mean low = .969), *F*(2, 61) = 2.44, *p* = .096. A parallel analysis performed on the response times showed no main effects, *F* < 1.9, and no interaction, *F* < 1.1.

Finally, the correlation between the Reading Span Task and the Semantic Anomaly Judgment Task was positive but not significant, *r*(64) = .21, *p* = .093, whereas in the Study 2 by Haarmann and his colleagues [3] it was *r*(60) = .32, *p* < .01. In addition, performance in cluster 1 of the Clustered Conceptual Span Task significantly predicted the score in both Reading Span and the Anomaly judgment task, whereas performance in cluster 2 and 3 predicted the score in the Reading Span task (see Table 4).

## Study 2

The findings from Study 1 provide good initial support for the newly constructed Spanish adaptation of the Conceptual Span task. Overall, the newly developed tasks showed excellent correspondence with the English versions of those tasks both as originally developed [3] and as further refined [14]. In addition, as expected, performance on the Clustered Conceptual Span Task significantly positively correlated with a Spanish adaptation of the Reading Span task, originally developed by Daneman and Carpenter [39], and with a measure of online semantic anomaly detection. The correlation of Clustered Conceptual Span with the semantic anomaly detection task was modest, however, and constrained by the near-ceiling levels of performance on the semantic anomaly task. Accordingly, in Study 2 we adopt a new and more challenging measure of text comprehension, that was available and normed for Spanish speaking participants.

From a theoretical perspective, it is notable that the magnitude of the correlation between Reading Span and the Clustered version of the Conceptual Span Task (*r* = .55, explaining 30% of the variance) was markedly stronger than the correlation between Reading Span and the Non-Clustered Span (*r* = .28, explaining only 8% of the variance). These results argue against a strong contribution of organizational abilities or WM to the relation between the Clustered Span measure and Reading Span, because for the Clustered version of the task little subjective organization during retrieval is required (for the reason that the semantic groupings for the items are themselves provided during the stimulus presentation).

In Study 2, in addition to administering both the Clustered and Non-Clustered Conceptual Span Tasks to a new and larger sample, we incorporated standardized measures of fluid reasoning and text comprehension, together with measures of verbal fluency (Controlled Oral Word Association Test), ideational fluency (Alternative Uses Task), speed of processing (Digit Symbol Substitution Test), and cognitive flexibility (Trail Making Test). We examined both the zero-order inter-correlations of these measures with the Clustered and Non-Clustered Conceptual Span Tasks, and the extent to which Clustered Conceptual Span predicted Reading Span when controlling for these other measures.

## Materials and methods

### Participants

One hundred and five Spanish-speaking student volunteers (79 women), with an average age of 19.87 (range 17–28, *SD* = 2.51) from the University of the Basque Country took part in the experiment. They had completed an average of 14.99 years (*SD* = 1.15) of formal education, and rated their subjective state of health as close to excellent on a 5-point scale ranging from 1 (*very poor*) to 5 (*excellent)*, *M =* 4.34, *SD* = 0.64; they also indicated on two 9-point scales that they were in a rather positive emotional state (1 *unhappy*, 9 *happy*), *M =* 6.53, *SD* = 1.75, and were moderately activated (1 *calm*, 9 *activated*), *M =* 5.17, *SD* = 1.43. They received monetary compensation for their participation.

### Materials

*Conceptual Span Task*. The same version as used in Study 1 was administered.

*Reading Span Task*. The same version as used in Study 1 was administered.

*Cattell’s Culture Fair Test* [47]. This task was used to assess fluid reasoning and includes visual tests that require the participant to perceive relationships between shapes and figures. In the present study Forms A and B of Scale 2 were used, each consisting of 4 subtests that require 3, 4, 3, and 2.5 min for completion: series, classification, matrices, and conditions. For example, in the *classifications* subtest, participants are shown 14 problems of five abstract shapes and figures and are asked to select which, out of five, does not match or belong with the others. The participant’s score was calculated summing all the correct answers produced within the time-limit.

*Text Comprehension* [48]. The Prolec-R battery is designed to evaluate and detect reading difficulties in students between 12 and 18 years old. The text comprehension subtest from Prolec-R employed in this study consisted of four written stories with four open-ended questions for each text. The participant read the text, listened to the questions and then answered them orally to the experimenter. The score on the text comprehension was defined as the number of questions correctly answered, with a maximum possible score of 16. The same rater scored all of the protocols, but one third (30%) of the protocols was scored by a second independent rater. Inter-rater reliability was very high, *r*(105) = .98.

*Trail Making test* [49]. This is a test for visual search speed, scanning, speed of processing, mental flexibility, as well as executive functioning. It consists of two parts in which the subject is instructed to connect a set of 25 dots on a sheet of paper as quickly as possible while still maintaining accuracy. In the first part, the targets are all numbers (1, 2, 3, etc.) and the test taker needs to connect them in sequential order; in the second part, the subject alternates between numbers and letters (1, A, 2, B, etc.). If the subject makes an error, the test administrator corrects them before the subject moves on to the next dot. The time required to finish the task in each part was taken into account, with analyses focused on the difference score (Part B minus Part A).

*Controlled Oral Word Association test* [50]. For the verbal fluency task participants were given 60 seconds to verbally generate as many Spanish words as possible, beginning with either the letter *F*, *A*, or *S*. Participants’ responses were audio taped (provided the participant consented to being recorded) to allow later crosschecking and confirmation of the experimenter’s written responses. Numbers, proper names, and variants of the same word (e.g., the same word with different suffixes, such as *thank* and *thankful*), as well as repetitions, were considered errors. The participant’s score for the COWAT was the total number of acceptable words generated for the three letters, but we also examined the number of errors (repetitions) produced. The same rater scored all of the protocols, but one third (30%) of the protocols was scored by a second independent rater. Inter-rater reliability was very high for both words generated, *r*(105) = .99, and repetitions, *r*(105) = .98.

*Digit Symbol Substitution test* [9]. This subtest was employed as a measure of speed of processing and working memory. It consists of 9 symbols matched with a corresponding numerical digit, and the participant is asked to match symbols with their corresponding digit. The number of matches made within 2 min is calculated.

*Alternative Uses Task* [51]. To assess ideational fluency a modified form of the Alternative Uses Task was administered, in which individuals are given the name of a common object (e.g., newspaper) and are asked to generate alternative, non-standard uses for the object (e.g., in addition to its common use as for *reading*, a newspaper also might be used to *start a fire*). The Alternative Uses Task used in this study included two phases. First, in the production phase, three common object items (chair, bed sheet, wooden pencil) were consecutively presented (on separate sheets); participants had 3 min per sheet to write down as many alternative uses for each object as possible that were both different from the common use and different from one another. An example was given before the task. Second, after completion of the production phase, following a procedure developed by Gilhooly, Fioratou, Anthony, and Wynn [52], participants were asked to review each of the uses they had generated and to indicate (by circling) those uses that they had first thought of while doing the task, that is, uses they had never seen or heard before, either in their own experience directly or in films, books, television, etc. The response protocols were scored using the task manual and new uses generated across the three objects were also calculated. The same rater scored all of the protocols, but one third (30%) of the protocols was scored by a second independent rater. Inter-rater reliability, calculated with the Pearson correlation coefficient, was very high, *r*(105) = .97.

### Procedure

All procedures were approved by the Ethics Committee/Board of the University of the Basque Country (UPV/EHU). The experiment began with the process of obtaining written informed consent, after which participants completed a brief demographic inquiry form. Then they were tested individually and performed eight tests, given in the same order: The Conceptual Span task, the Reading Span task, the Cattell Culture Fair test, the Reading Comprehension subtest from Prolec-Se, the Trail Making test, the Controlled Oral Word Association test, the Symbols Substitution task from WAIS, and the Alternative Uses Task. All words and sentences in the Conceptual span task and the Reading span task were visually presented using software E-prime 2.0 [45], in order to present stimuli in a controlled manner and visual format. The experimental session lasted approximately 80 minutes.

### Results and discussion

For all statistical analyses, the significance level was set at *p <* .05, unless otherwise noted.

### Performance on the tasks

Table 5 shows mean performance for each of the tasks.

**Table 5:** Mean performance on the measures employed in Study 2.

Measures	Mean	*SD*	Min	Max	Maximum possible
**Clustered Conceptual Span**	44.07	5.83	31	57	64
**Cluster 1**	14.45	2.74	6	21	24
**Cluster 2**	16.25	2.71	7	20	24
**Cluster 3**	13.37	2.01	11	16	16
**Non-Clustered Conceptual Span**	31.58	4.63	21	44	48
**Reading Span**	4.07	1.19	2	6	6
**CCF**	35.54	3.86	27	45	46
**Text comprehension (Prolec-R)**	13.66	1.53	10	16	16
**Trail Making Test**					-
Trail Making Test **Part A**	23.31	7.93	9	53	
Trail Making Test **Part B**	49.23	17.32	22	101	
**COWAT**	54.24	11.84	25	92	-
COWAT **Repetitions**	0.50	0.87	0	5	
**Digit Symbol**	84.68	11.87	60	117	133
**AUT**	18.18	5.58	6	37	-
**AUT New**	6.92	4.23	1	22	-

On the key clustered version of the Conceptual Span Task participants recalled, on average, a proportion of .69 (44.07 words, out of 64 words maximum; range 31–57, *SD* = 5.83), and in the Non-Clustered version they recalled a proportion of .66 (31.58 words, out of 48 words maximum; range 31–44, *SD* = 4.63). Thus, participants had a higher proportion of words recalled in the Clustered than in the Non-Clustered version of the Conceptual Span task, *t*(104) = 3.40, *p* = .001. These levels of performance are very similar to those reported by [3] with 64 and 66 participants. Using the same method to calculate an estimate of semantic STM capacity as was described earlier for Study 1, we found an average STM capacity of 4.1 for Clustered Conceptual Span and 3.7 for Non-Clustered Conceptual Span. Spearman-Brown split-half reliability for odd-numbered versus even-numbered trials was acceptable for the Clustered Span task (.72) but lower for the Non-Clustered Span (.52 for odd-numbered vs. even-numbered trials, .63 for first half vs. second half).

On the Reading Span Task, participants' mean score was 4.07 (maximum score was 6; range 2–6, *SD* = 1.19). This score is also very similar to the score reported by [3], with 60 young adults (*M* = 3.93, *SD* = .84).

On the Cattell Culture Fair test of fluid reasoning, participants' mean score was 35.54 (maximum score was 45; range 27–45, *SD* = 3.86).

On the Reading Comprehension subtest from Prolec-Se, the mean score was 13.66 (maximum score was 16; range 10–16, *SD* = 1.53).

On the Trail Making test, participants’ mean score for part A was 23.31 seconds (range 9–53, *SD* = 7.93), and for part B, mean score was 49.15 (range 22–92.98, *SD* = 17.11). On the Trail Making Test part B, the scores for 4 participants were outliers; we replaced their scores with values that were 3 SD from the sample mean, after their exclusion. The average difference score (part B minus part A) was 25.84 (range 4.00–76.98, *SD* = 16.08).

On the Controlled Oral Word Association Test, the mean score was 54.24 (range 25–92, *SD* = 11.84). Most participants made few errors (mean error = 0.50, range 0–4, *SD* = 0.82, one outlier value of 5 replaced with a value of 4).

On the Symbols Substitution task from the WAIS, participants' mean score was 84.68 (maximum score was 117; range 60–117, *SD* = 11.93).

Finally, on the Alternative Uses Task, the mean score was 18.18 (range 6–37, *SD* = 5.58); among these responses, participants indicated that an average of 6.92 of their responses (range 1–22, *SD* = 4.23) were newly generated during the experimental task rather than recollected from previous experiences or observations.

### Correlations between measures

Table 6 shows the product moment correlations between the measures. The Clustered and Non-Clustered versions of the Conceptual Span Task strongly positively correlated with each other, *r*(105) = .52, *p* < .001.

**Table 6:** Pearson correlations between measures employed in Study 2.

	Reading Span	Prop. Clustered	Prop. Non-Clustered	AUT	AUT New	CCF Total	Symbols	Reading Compr.	COWAT	COWAT Repetition	Trails A–B
Reading Span	1.00	.34**	.25**	–.01	–.08	.17	.09	.10	.11	–.20*	–.07
Prop. Clustered		1.00	.52**	–.05	.16	.26**	.22*	.07	.16	–.13	–.06
Prop. Non-Clustered			1.00	.02	–.03	.26**	.22*	.19*	.12	–.16	–.06
AUT				1.00	.57**	.06	.14	.20*	.36**	.02	.02
AUT New					1.00	–.09	–.01	.03	.15	–.02	–.05
CCF Total						1.00	.18	.28**	.30**	.00	–.20*
Symbols							1.00	.10	.29**	–.16	–.20*
Reading Compr.								1.00	.26**	.04	.00
COWAT									1.00	.10	–.11
COWAT Repetition										1.00	.11
Trails A–B											1.00

**p < .01 (2-tailed).

*p < .05 (2-tailed).

As predicted, there was a significant positive correlation between performance on the Reading Span Task and the average score on the Clustered Conceptual Span Task, *r*(105) = .34, *p* < .001, and with the scores on the Non-Clustered Conceptual Span Task, *r*(105) = .25, *p* = .018.

Paralleling the findings reported in [14], both Clustered and Non-Clustered Conceptual Span positively correlated with fluid reasoning performance, as assessed by the CCF. These correlations (now within young adults alone, rather than across younger and older adults) were essentially the same magnitude for the Clustered Conceptual Span task (*r* (105) = .26, *p* < .01; *r* (36) = .22, *p* = .21, in 2010 study), but lower than in the earlier study for the Non-Clustered version (*r* (105) = .25, *p* < .01; *r* (36) = .55, *p* = .001, in [14]). It seems that the Aizpurua and Koutstaal [14] correlation, given the smaller sample size of 36 younger adults, may have been an overestimate of the correlation between fluid reasoning performance and Non-Clustered Conceptual Span performance. Alternatively, or perhaps in addition, the correlation in Study 2 may have been somewhat reduced as a result of participant fatigue effects. In Study 2, the Non-Clustered version was always administered after the Clustered version and, unlike in Aizpurua and Koutstaal [14], where the two versions of the Conceptual Span task were administered in separate testing sessions, in Study 2, participants completed both tasks in a single session. This may also partially explain the lower internal reliability we observed for the Non-Clustered span task in Study 2 than in Study 1. More importantly, across both the previous [14] and the current study, the purer measure of Clustered Conceptual Span was found to consistently explain between 5 to 7 percent of the variance in the fluid reasoning performance of younger adults.

### Correlations with reading span, controlling for other cognitive measures

We examined the correlation between the key Clustered conceptual span measure and Reading Span when separately controlling for standardized measures of fluid reasoning (CCF), text comprehension, verbal fluency (Controlled Oral Word Association Test), ideational fluency (Alternative Uses Task), speed of processing (Digit Symbol Substitution Test), and cognitive flexibility (part B minus part A for the Trail Making Test). The partial correlation of conceptual span with Reading Span remained significant when controlling for each of these measures: fluid reasoning (partial *r* = .31, *p* = .001), text comprehension (partial *r* = .34, *p* < .001), verbal fluency, correct responses (partial *r* = .33, *p* = .001), verbal fluency, repetition errors (partial *r* = .32, *p* = .001), ideational fluency, total uses (partial *r* = .34, *p* < .001), ideational fluency, newly generated uses (partial *r* = .36, *p* < .001), speed of processing (partial *r* = .33, *p* = .001), and cognitive flexibility (partial *r* = .34, *p* < .001).

Consistent with these findings, a multiple linear regression analysis predicting Reading Span when including all of the variables assessed in Study 2 (proportion in Clustered Conceptual Span, proportion in NonClustered Conceptual Span, AUT, AUT New, Total CCF, Symbols, Comprehension, COWAT, COWAT Repetitions, and Trails A minus B), revealed that Clustered Conceptual Span was the only significant predictor of Reading Span, standardized ß = .34, *t* = 2.81, *p* = .006. In addition to Clustered Conceptual Span, the number of repetition errors on the COWAT––arguably also an indication of online semantic processing and maintenance––was also a negative predictor of Reading Span (standardized ß = -.17, *t* = -1.75, *p* = .08), *F*(2, 102) = 8.42, *p* < .001, R^2^ = .14. In contrast, as can be seen from Table 7, Non-Clustered Conceptual Span added little predictive value over and above that provided by Clustered Conceptual Span.

**Table 7:** Summary of multiple linear regression model predicting Reading Span (R^2^ = .18).

Variable	β	t-value	Sig.
Proportion in Clustered	.34	2.81	.006**
Proportion in Non-Clustered	.04	0.31	.76
AUT	.12	0.92	.36
AUT New	-.21	-1.72	.09
CCF (Total)	.02	0.18	.86
Symbols	-.06	-0.61	.55
Comprehension	.04	0.43	.67
COWAT	.05	0.46	.65
COWAT Repetitions	-.17	-1.75	.083
Trails (A minus B)	-.05	-0.47	.64

**p < .01.

AUT = Alternative Uses Task; CCF = Cattell Culture Fair; Comprehension = Reading Comprehension subtest from the Prolec-Se; Symbols = Digit Symbol Substitution Test; COWAT = Controlled Oral Word Association Test; Trails (A minus B) = Trail Making Test.

Despite the finding that Clustered Conceptual Span strongly correlated with Reading Span, Clustered Conceptual Span did not correlate with the newly introduced measure of Text Comprehension, even though performance was not at ceiling on the comprehension task. Instead, as can be seen from Table 6, only Non-Clustered Conceptual Span significantly positively correlated with the Text Comprehension measure (*r* = .19, *p* = .047) and the Text Comprehension measure, in turn, correlated with more strategically-based problem-solving measures, including Fluid Reasoning (*r* = .28, *p* = .004), and AUT (*r* = .20, *p* = .04).

## Conclusions

This study newly constructed, tested, and validated a Spanish adaptation of the Conceptual Span task––developing both a Clustered and Non-Clustered version of the task––to allow efficient assessment of individual differences in the semantic component of STM in Spanish speakers. Across two studies, the newly developed tasks showed excellent correspondence with the English versions of those tasks both as originally developed by Haarmann and his colleagues [3], and as further refined by Aizpurua and Koutstaal [14].

Replicating the finding from Study 1, Clustered Conceptual Span was a numerically stronger predictor of Reading Span than was Non-Clustered Conceptual Span. Additionally, the correlation between Clustered Conceptual Span and Reading Span remained essentially intact when controlling for each of several other cognitive variables, including fluid reasoning, text comprehension, verbal and ideational fluency, speed of processing, and cognitive flexibility. Therefore, the ability to maintain, update and manipulate conceptual information in STM constitutes a significant and uniquely powerful contributor to successful sentence processing and language comprehension. However, whereas Clustered Conceptual Span was strongly and consistently associated with Reading Span, Non-Clustered Conceptual Span appeared–in Study Two–to be a closer correlate of text comprehension, a task that placed stronger demands on drawing inferences and problem-solving. It is possible that the contribution of executive processes to the reading comprehension task masked the sentence comprehension component involved in reading comprehension.

Previous research has most often used a Non-Clustered version of the conceptual span task (e.g., [53] Dutch translation for children; Experiments 1 and 2 of [3]; [15]). As noted in the Introduction, the Non-Clustered version requires additional on-line re-organization of the stimuli as the participants must first identify, and then themselves actively group (i.e. "cluster") the relevant items from the designated category. Yet, as earlier noted by Haarmann and his colleagues [3] while this re-organization may itself draw on semantic STM, "clustering ability may also rely on factors that are independent of semantic STM capacity. Therefore, we recommend use of the clustered span task to index semantic STM capacity in future studies" (p. 338)

In accordance with these considerations, in the two studies reported here we have presented comparisons of the clustered versus Non-Clustered tasks, but have always prioritized the clustered task results as the more informative and pure measure of semantic STM and so, for example, administered the Clustered task first. Notably, however, the materials we developed, normed, and offer here can––depending on a particular researcher's needs––be adapted and used for *either* clustered or Non-Clustered task administration. For example, the materials given in the S1 File could be presented in a way to allow for *alternate forms* of a clustered task, as might be desirable if repeatedly assessing participants' semantic STM performance across a research study, or in a neuropsychological context so as to assess a patient's semantic STM before and after a rehabilitation treatment intervention. Given that some patients demonstrate impairments predominantly in phonological STM and other patients show impairments primarily in semantic STM, rehabilitation treatments specifically designed to improve phonological versus semantic aspects of STM may lead to both generalized and *type-specific* enhancements in sentence comprehension.

For example, Harris and colleagues recently performed a memory rehabilitation study [26] with two older patients who, following a cerebral vascular accident, showed contrasting impairments in phonological STM versus semantic STM. Both interventions involved one 1.5 hour per session per week, over 10 weeks, in combination with at-home exercises. However, whereas the phonologically-based intervention required recognition of previously presented non-words, with each training list being one item above the patient's span and examiner feedback given after each trial, the semantically-based intervention used lists of real words, and the patients were encouraged to think about the meaning of each word as it was presented. Despite the fact that the treatment materials were confined to lists of words, the patient with impaired semantic STM showed significant improvements in sentence anomaly judgment following the semantic treatment intervention, but not after the phonologically-focused intervention, whereas the patient with impaired phonological STM showed post-treatment improvement on sentence repetition. Harris, Olson, and Humphreys [26] underscore the need for more research into STM rehabilitation, not only because of the high incidence of memory deficits following brain injury, but also because such interventions might lead to generalized treatment effects to other abilities (e.g., from word lists to sentences) and provide new insights into the processes supporting STM.

Although measures of semantic STM can be criticized on the grounds that they may simply reflect an individual's semantic knowledge, a number of considerations argue against vocabulary differences strongly contributing to the current outcomes. First, the stimuli used in the Conceptual Span task were all chosen–based on normative data–to likely be familiar to participants. Second, following procedures adopted in earlier studies using the Conceptual Span task, all participants were explicitly familiarized with the items in each of the categories before administration of the Conceptual Span tasks. Third, although we did not include a separate assessment of vocabulary, in Study 2 we did include the Controlled Oral Word Association Test (COWAT)––a measure of verbal fluency. Performance on this verbal fluency task did not strongly correlate with performance on either the Clustered (*r* = .16) or NonClustered (*r* = .12) Conceptual Span tasks, even though the COWAT did significantly correlate with Reading Comprehension (*r* = .26, *p* < .01). Taken in combination, these considerations suggest that, although there may be a contribution of differences in semantic knowledge or vocabulary to all measures of semantic STM, those contributions were minimized in the current research.

From a broader perspective, our development of a Spanish adaptation of the conceptual span tasks may help to focus additional research on the important, but until now comparatively still under-investigated question of how people temporarily hold in mind––and keep active––semantic information. Increased attention to the role of short-term semantic memory in cognitive processing also coheres well with recent efforts to better integrate concepts relating to STM storage with goal-related processing, awareness, and attention [54], and to findings underscoring individual differences in reliance on phonological processing for the interfacing of semantic and abstract information in ongoing thinking and problem solving (e.g., deaf signing individuals, [55]). It is hoped that the Spanish adaptation of the Clustered and Non-Clustered Conceptual Span tasks that we have here provided contribute to our understanding of a ubiquitous but under-researched aspect of human cognition that guides and enables nearly all complex activities: our ability to temporarily maintain, and flexibly draw upon, the meanings of words and things.

## Supporting information

S1 File Categories and exemplars for the clustered and non-clustered versions of the conceptual Span Task. (PDF)
