# Supplementary material for: A new index of semantic short-term memory: Development and validation of the conceptual span task in Spanish
Source: PLoS One. 2018 Dec 27;13(12):e0209368. doi: 10.1371/journal.pone.0209368 (PMC6307978; doi:10.1371/journal.pone.0209368)
Supplement: S1 File — (PDF) [file pone.0209368.s001.pdf]

S1 File Categories and exemplars for the Clustered and Non-Clustered versions of the Conceptual Span Task.

Clustered version

**AVES (BIRDS)**	águila, gorrión, loro, canario, gaviota, avestruz, halcón, cigüeña
**FRUTAS (FRUITS)**	pera, naranja, plátano, melón, fresa, kiwi, cereza, sandía
**ROPA (CLOTHES)**	camisa, falda, calcetín, chaqueta, jersey, abrigo, vestido, blusa
**PARIENTES (RELATIVES)**	tío, abuelo, primo, hermano, madre, sobrino, nuera, hijo
**CLIMA (WEATHER)**	nieve, granizo, tormenta, huracán, viento, sol, tornado, trueno
**METALES (METHALS)**	plata, oro, cobre, acero, bronce, plomo, cinc, platino

Non-Clustered version

**INSECTOS (INSECTS)**	mosquito, abeja, hormiga, avispa, pulga, piojo, gusano, polilla
**VERDURAS (VEGETABLES)**	acelgas, coliflor, vainas, puerros, berza, cebolla, brócoli, col
**CUERPO (BODY PARTS)**	pierna, cabeza, pie, mano, ojo, dedo, nariz, oreja
**TIEMPO (TIME UNITS)**	hora, minuto, año, mes, día, semana, siglo, década
**ÁRBOLES (TREES)**	roble, manzano, peral, abeto, castaño, haya, cerezo, naranjo
**TIERRA (EARTH)**	río, valle, volcán, cabo, golfo, lago, mar, sierra
